# Malaria control in South Sudan, 2006–2013: strategies, progress and challenges

**DOI:** 10.1186/1475-2875-12-374

**Published:** 2013-10-27

**Authors:** Harriet Pasquale, Martina Jarvese, Ahmed Julla, Constantino Doggale, Bakhit Sebit, Mark Y Lual, Samson P Baba, Emmanuel Chanda

**Affiliations:** 1 Ministry of Health, National Malaria Control Programme, Juba, Republic of South Sudan; 2 Population Services International, Juba, Republic of South Sudan

**Keywords:** Malaria control, Policy and strategy, Collaboration, Capacity building, Monitoring and evaluation

## Abstract

**Background:**

South Sudan has borne the brunt of years of chronic warfare and probably has the highest malaria burden in sub-Saharan Africa. However, effective malaria control in post-conflict settings is hampered by a multiplicity of challenges. This manuscript reports on the strategies, progress and challenges of malaria control in South Sudan and serves as an example epitome for programmes operating in similar environments and provides a window for leveraging resources.

**Case description:**

To evaluate progress and challenges of the national malaria control programme an in-depth appraisal was undertaken according to the World Health Organization standard procedures for malaria programme performance review. Methodical analysis of published and unpublished documents on malaria control in South Sudan was conducted. To ensure completeness, findings of internal thematic desk assessments were triangulated in the field and updated by external review teams.

**Discussion and evaluation:**

South Sudan has strived to make progress in implementing the WHO recommended malaria control interventions as set out in the 2006–2013 National Malaria Strategic Plan. The country has faced enormous programmatic constraints including infrastructure, human and financial resource and a weak health system compounded by an increasing number of refugees, returnees and internally displaced people. The findings present a platform on which to tailor an evidence-based 2014–2018 national malaria strategic plan for the country and a unique opportunity for providing a model for countries in a post-conflict situation.

**Conclusions:**

The prospects for effective malaria control and elimination are huge in South Sudan. Nevertheless, strengthened coordination, infrastructure and human resource capacity, monitoring and evaluation are required. To achieve all this, allocation of adequate local funding would be critical.

## Background

In 2010, about 219 million malaria cases and 660,000 deaths were reported globally. The disease remains a major cause of morbidity and mortality, exacting its greatest toll in sub-Saharan Africa where over 80% of cases and 90% of deaths occur [1]. The huge burden could be ascribed to efficient afro-tropical malaria vectors with strong vectorial capacities that maintain high levels of transmission. As well as, environmental factors and climatic changes, population movement, deteriorated socioeconomic situation, lack of access to effective anti-malaria treatment and use of fake anti-malarial drugs [2]. With increasing international funding, malaria endemic countries have stepped up control efforts at individual and community levels [3]. Interventions include; early diagnosis with rapid diagnosis test (RDTs), treatment with artemisinin-based combination therapy (ACT), indoor residual spraying (IRS), long-lasting insecticidal-nets (LLINs) and intermittent preventive treatment (IPTp) [1]. However, in post- conflict African environments, effective control has remained a daunting undertaking due to a multiplicity of challenges including high malaria transmission intensities.

South Sudan has borne the brunt of years of chronic liberation warfare from the time of Sudan’s independence from Anglo-Egyptian rule in 1956 [4]. The war destroyed physical infrastructure, social structures and virtually collapsed the health system. During the last phase of the conflict (1983–2005), international donors, non-governmental organizations (NGOs) and faith-based organizations (FBOs) assumed responsibility for basic health service delivery and helped build nascent health institutions [5]. The hostilities ended with the signing of the Comprehensive Peace Agreement (CPA) in January 2005. Following the referendum in January 2011, South Sudan’s independence was proclaimed on July 9, 2011 as a sovereign new nation and marking its secession from Sudan. The country’s public health systems remain devastated from the legacy of violence and instability, with effective health services still low at under 25% [4]. With attainment of independence, there have been deliberate efforts to shift from fragile or post-conflict top-bottom systems centered on emergency relief and primary health care administered by international NGOs to more sustainable development systems managed by the Ministry of Health (MoH) in South Sudan [5]. More than 80% of healthcare available is still provided by international NGOs.

Upon the signing of the CPA, South Sudan has been characterized by enormous infrastructure, human and financial resource constraints and a weak health system against a huge burden of diseases [6]. The country has one of the highest malaria burdens in sub-Saharan Africa. Improved health care delivery by the MoH has facilitated for the planning, coordination, implementation and monitoring of malaria control interventions. South Sudan was successfully awarded the Global Fund rounds 2, 7 and 10 for malaria control and was successful in obtaining financial support from other funding agencies to scale-up interventions [7]. A growing body of evidence demonstrates that rational malaria control and prevention significantly reduce illness and death and thus contributing directly to the attainment of health-specific Millennium Development Goals (MDGs). However, through 2012 malaria remained endemic in all of the country’s 10 administrative states [8].

The malaria control situation is threatened by the impact of refugees, returnees, internally displaced populations, and natural disasters, i.e. flooding, that put added strain on an already weakened system from years of conflict and that may destabilize whatever gains that have been made. Given gross constraints to access and the austerity budget announced by the government, humanitarian need remains very high. It is estimated that out of a projected population of 11.1 million people, 40% of the population (4.5 million people) are in urgent need of humanitarian assistance [9]. While South Sudan is in the post-conflict phase, some volatile states of the country are experiencing active conflict with armed hostilities and inter-communal violence persisting and displacing tens of thousands of people and continue to threaten development efforts and humanitarian aid by UN agencies, IOM and NGOs. The challenging operational environment of South Sudan continues to require emergency response and protection, increased support for livelihoods and resilience, and strong coordination [9].

To reduce the malaria burden, the World Health Organization (WHO) recommended case management and vector control tools have been implemented expansively in South Sudan [10]. To be able to assess programme implementation and progress towards attainment of MDGs, measuring the impact of malaria control on reducing disease morbidity and mortality is essential [11]. The MoH has a well-established and functioning routine information system through the Integrated Disease Surveillance Response (IDSR) and the national Health Management Information System (HMIS) including sentinel site surveillance to regularly monitor the outcomes of malaria control [12]. Over seven years (2006 – 2013) of implementation of recommended interventions, the country has experienced marked heterogeneity in effectiveness of malaria control efforts [8]. This manuscript reports on the strategies, progress and challenges of malaria control in South Sudan and is envisioned to save as an archetype for similar environments and a window for leveraging resources.

## Case description

In March 2012, South Sudan undertook an in-depth review of the national malaria control programme (NMCP) [13]. The decision was made in the context of an observed increase in malaria incidence and deaths in the country. The review aimed at strengthening strategic planning and to inform resource mobilization for scaling up delivery of malaria control services. The findings are critical for informing the development of the 2014–2018 national strategic plan for malaria control.

### Geography and population

**Figure 1:**
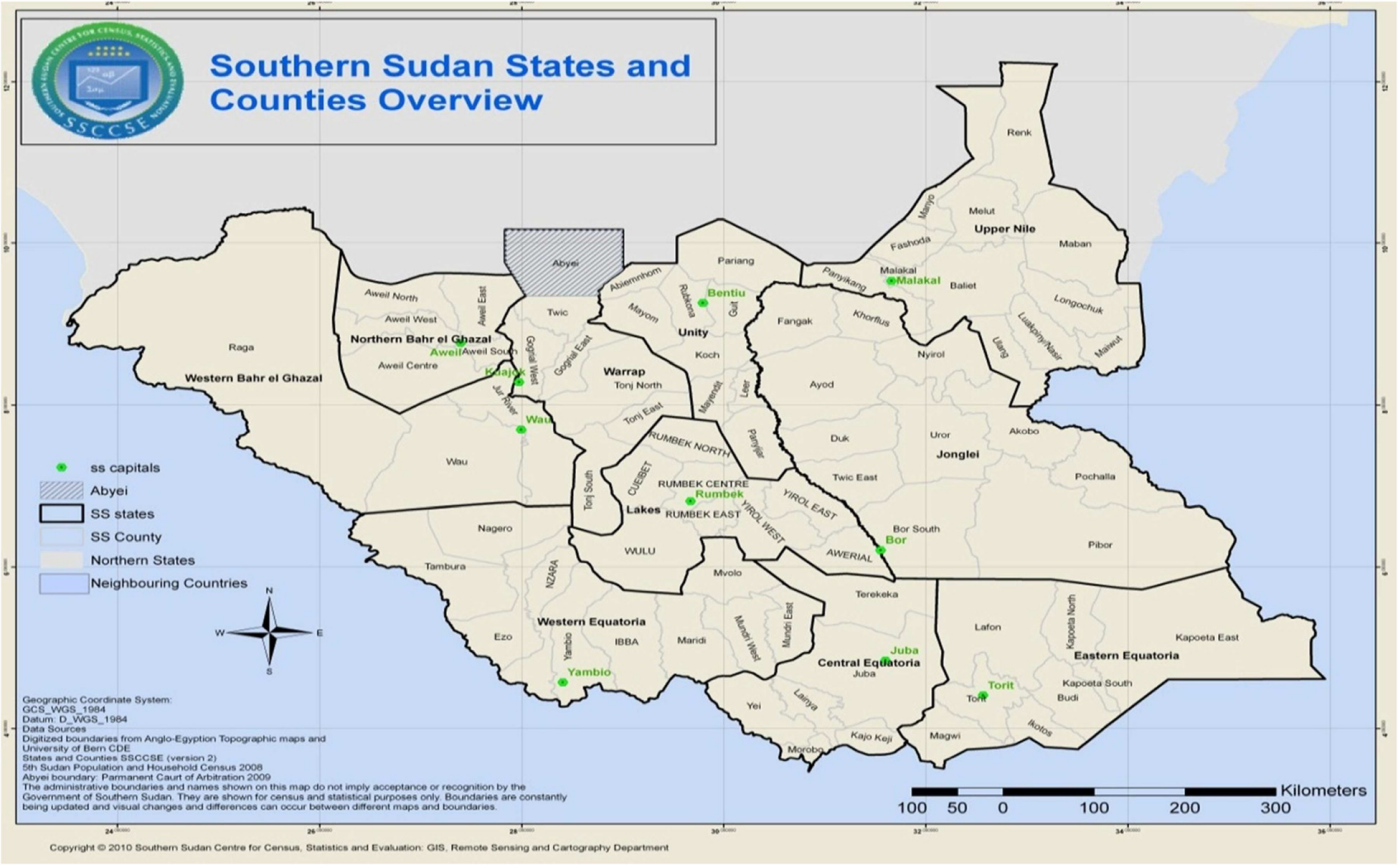
Map of South Sudan showing state boundaries.

South Sudan is a land-locked country in East Africa, bordering six malarious countries: Central African Republic in the west, Democratic Republic of Congo in the southwest, Ethiopia in the east, Kenya and Uganda in the south and Sudan in the north, (Figure 1). The country covers an area of approximately 650,000 km^2^ of land mass, between 8° and 18° degrees south latitude and between 20° and 35° degrees east longitude, divided into 10 states with a total population of about 8.3 million [14]. The states are the basic planning levels for health service delivery. The climate is tropical with average temperatures ranging between 20°C and 37°C and relative humidity between 26% and 88%. Annual rainfall ranges between 1,000 mm in the South and 400 mm in the northern parts. Similarly, the duration of the rainy season varies from 7–8 months in the South to 5–6 months in the northern region. Malaria is endemic across the entire country with year-round transmission but peaking towards the end of the rainy season from September to November [8].

### Evaluation of malaria control progress and challenges

A comprehensive assessment was undertaken according to the WHO standard procedures for Malaria Programme Review (MPR) [15]. The evaluation process was conducted in 4 phases; 1) developing an action plan and organizing various stakeholders and partners to agree on the objectives, 2) thematic desk reviews of national documents and other relevant sources and the selection of tools for field evaluation; 3) joint analysis of thematic reports by internal and external reviewers and field visits to validate thematic reports, and 4) report writing and planning for implementation of the recommendations. Thematic review groups for key malaria programme areas comprised internal reviewers from the NMCP and country Roll Back Malaria (RBM) partners. Routine surveillance data from the HMIS, data from population-based household surveys and various operations research reports were retrospectively analyzed. The findings of thematic internal desk review were triangulated through field visits by internal and external review teams. States were considered as the primary sampling unit. Three teams of five people each were formed for field visits to randomly sampled states in Central Equatoria, Western Bahr el Ghazal and Upper Nile. To ensure completeness, thematic review reports were updated with information on key findings from the field.

### Malaria epidemiology in south Sudan

#### The malaria risk

**Figure 2:**
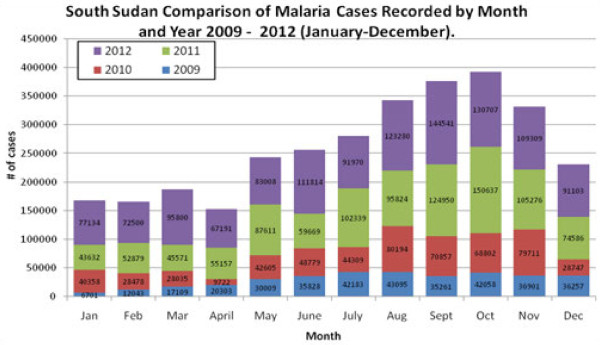
Regional variation of malaria.

**Figure 3:**
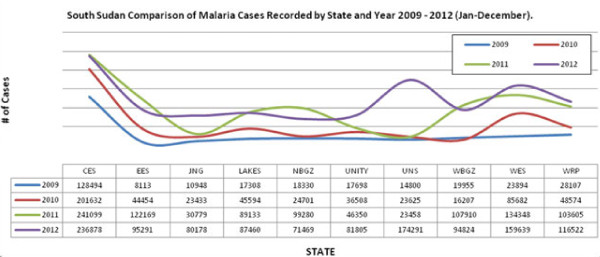
Distribution of malaria cases by state 2009 – 2012.

**Figure 4:**
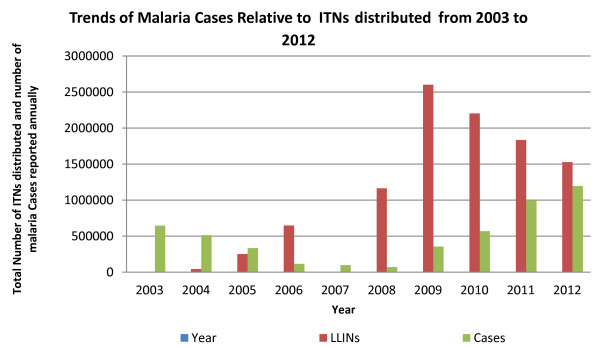
Trends of total malaria cases relative to LLIN distributed from 2003 to 2012 (Source: HMIS/DHIS).

**Figure 5:**
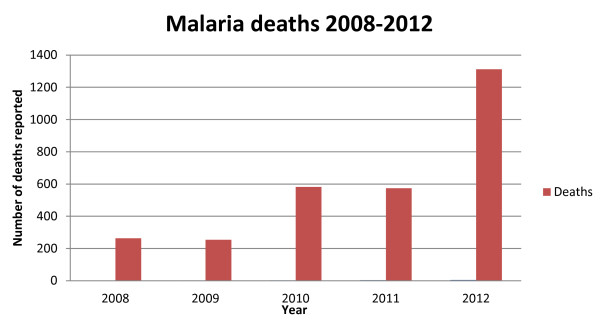
Trends of malaria deaths from 2008 to 2012 (Source: HMIS).

Malaria is the leading cause of morbidity and mortality in the country, accounting for 20% to 40% morbidity with over 20% of deaths reported at health facilities and 30% of all hospital admissions [8]. The disease is endemic country-wide putting the entire population at risk of infection and exacting a greater toll in children under five and pregnant women. Malaria endemicity varies from hypo-endemicity, through meso-endemicity, hyper-endemicity to holo-endemicity. Parasite prevalence ranges from less than 1% to more than 40% with great variability across the states and is higher in rural areas than in urban areas [8]. The HMIS data indicate a gradual increase in the number of cases and deaths due to malaria as reported by health facilities between 2008 and 2012 (Figures 2, 3, 4 and 5).

#### Malaria parasites and vectors

*Plasmodium falciparum*, the most virulent parasite species is dominant and responsible for up to 94% of all morbidity, 5% is caused by *Plasmodium vivax*, 0.7% is due to *Plasmodium malariae*, and mixed infections occur in 6.3% of cases especially in the Greater Equatoria region [8]. The major vectors are *Anopheles gambiae s.s., Anopheles arabiensis* and *Anopheles funestus*, with *Anopheles nili* as a secondary vector but little is known about their relative distribution in time and space [2].

#### Temporal and spatial distribution of malaria

The eco-epidemiological profile of South Sudan is ideal for proliferation of malaria vectors and country-wide perennial malaria transmission with seasonal variations. Malaria transmission season is longer in the southern than in the northern regions and peaks towards the end of the rainy season in September to November [8].

#### Anti-malarial drug efficacy and resistance

Anti-malarial drug efficacy studies for chloroquine (CQ) and sulphadoxine-pyrimethamine (SP) have been conducted across the three greater regions of the country between 2001 and 2003. High levels of *Plasmodium falciparum* resistance to CQ and SP were found ranging from 40% to 93% for CQ and 15% to 69% for SP, and the country switched its treatment policy to ACT [13]. Follow up studies are being conducted in randomly selected sites in 2013 [16].

#### Insecticide resistance in malaria vectors

There is very limited data on insecticide resistance in malaria vectors in South Sudan. The only available information is on insecticide susceptibility of *Anopheles* species populations to DDT and deltamethrin in four localities of Juba County, Central Equatoria State. According to the WHO criteria, susceptibility was detected in Bari and Juba payams and suspected resistance in Munuki and Kator payams to 4% DDT, resistance was detected in Bari payam, and suspected resistance in Munuki, Katour and Juba payams to 0.05% deltamethrine [17]. The study did not characterize the *Anopheles* species to identify malaria vectors. Insecticide resistance monitoring and surveillance system is being established.

### Malaria control in south Sudan

#### Overview

The MoH through the NMCP is responsible for planning, coordinating, implementing and monitoring of malaria control interventions. While the NMCP in South Sudan is relatively young, the malaria control policy and strategic framework is well defined, with key WHO recommended interventions being scaled up and monitoring and evaluation systems established (Table 1). A national strategic plan for malaria control was developed for the period of 2006–2013 with several malaria technical guidelines and tools to operationalize the plan. Malaria control is well articulated in the National Development Agenda, National Health Sector Strategic Plan (HSSP) and the 2012–2016 Health Sector Development Plan (HSDP) [4]. The HSSP prioritizes malaria control and prevention and endeavours to attain universal coverage with cost effective malaria interventions. Malaria is a key component of the basic package of health services and both curative and preventive interventions are delivered at all health system levels, including the community [4]. The HSDP reflects the political will of the sovereign government of South Sudan to streamline and transform the weak health system, thus creating a platform for tailoring effective malaria control.

**Table 1:** Chronology of key milestones of the NMCP over the years: 1998-2013

**Year**	**Key milestone(s)**
1998	WHO begins to support the coordination and management of malaria control within the Southern Sudan Health Secretariat.
1999	WHO established an EWARN to facilitate rapid reporting and investigation of suspected outbreaks by a network of NGO-operated health facilities operating in southern Sudan.
2003	A Malaria Task Force was formed in order to allow a broad discussion and consensus building mechanism among partners with respect to the new malaria treatment policy.
2004	The national Malaria Control Programme is formed as part of the Secretariat for Health, then based in Nairobi.
2005	Vector control needs assessment for IVM was done in 2005 following the Resolution (EM/RC.52/R.6).
	USAID began to support disease surveillance activities in southern Sudan with funding through the CDC as a component of the Sudan Health Transformation Project (SHTP I).
2006	The Secretariat inclusive of NMCP was relocated to Juba, South Sudan; throughout this time and beyond, the NMCP was staffed by one person, the Programme Manager.
	USAID through MSH seconded one full time malaria Technical Advisor to the NMCP to support the Programme Manager and team.
	First IDSR Task force formed and endorsed case definitions for a small set of priority diseases.
2007	The first Monitoring and Evaluation Officer was recruited with support from USAID through MSH.
	NMCP Office established and the first Malaria Prevention and Control Strategic Plan (July 2006-June 2011, extended to 2013) was finalized with support from USAID funded Technical Assistance.
	The ACT based treatment Policy was finalized leading to development of the first Malaria Treatment Policy; This was followed by a roll out of training of health workers in all the health facilities between 2007 and 2010.
	The Country Malaria Technical Working Group was formed to ensure coordinated malaria programming. The TWG has played a critical role in supporting NMCP to fulfill its functions.
	NMCP drafted a concept paper advocating for mass distribution campaigns to rapidly increase LLIN coverage.
	The first African Malaria Day was commemorated on April 25th 2007. These are now commemorated annually as World Malaria Days.
2008	LLIN mass campaigns piloted in 3 states with MDTF and USAID support; since then Mass LLIN distribution campaigns have been rolled out in all the states.
	WHO takes on the IDSR mantle with assistance from USAID and ECHO.
2009	The GoSS recruited 3 Public Health Officers for Vector Control, Case Management/BCC and M & E.
	IDSR Action Plan 2009–2013 was completed.
	The HMM program rolled out to further increase access to ACTs.
	The first MIS conducted with support from partners and a malaria epidemiological map developed.
2011	The NMCP Manager recruited alongside the Case Management and Monitoring and Evaluation Specialist with support from the GFATM; State Malaria Coordinators recruited with Government support.
	The first annual malaria planning and review meeting held with state malaria coordinators and M&E officers.
	South Sudan becomes a WHO member state, the 23rd under EMRO.
2012	With support from its partners NMCP established 32 sentinel which are used for monitoring malaria intervention coverage.
	Vector control Specialist/Medical Entomologist- consultant recruited.
	The first vector control conference held with state Director Generals, malaria coordinators and M&E officers.
	Recommendations on addressing malaria vector control challenges published- Chanda *et al.*, 2013.
2013	The Malaria Programme Review process and follow-up MIS concluded.

### Programme intervention areas

#### Malaria vector control

Historically, vector control was operationally harnessed for malaria prevention in South Sudan. In the late 70s and early 80s IRS and larviciding were implemented by the local vector control units to prevent malaria transmission in and around the major towns and municipalities. However, due to the collapse of infrastructure and public services these interventions stopped in 1983 and are currently not available at operational level [10]. With the return of peace in the country, the WHO-led integrated vector management (IVM) has been adopted as the main approach for vector control. The NMCP developed a draft strategic plan for IVM for the period 2007–2012, a national policy and an implementation plan for IVM [18]. The approach is to consolidate the use of LLINs while introducing additional interventions, i.e. IRS and larval source management (LSM), where applicable. Presently, the distribution of LLINs remains the only key operational vector control intervention with limited use of IRS and larviciding by Mentor Initiative, an NGO in Malakal County [19]. To date over 9.0 million LLINs have been distributed through mass distribution campaigns and health facility based routine distribution. The NMCP is putting in place implementation arrangements for operational deployment of targeted IRS and larviciding.

#### Malaria in pregnancy

The recommended channel for delivering Malaria in pregnancy (MIP) interventions is through comprehensive and focused ante-natal care (ANC) services with a three-pronged package; effective treatment of malaria and anaemia, IPT and use of LLINs [10]. According to the national guidelines for malaria management in pregnancy, all pregnant women attending ANC services should receive at least two doses of SP spaced at least one month apart as directly observed treatment and at least three doses to women infected with HIV. To enhance the uptake free LLIN are distributed during ANC visits and all pregnant women are encouraged to use the nets. A collaborative effort involving the NMCP, Reproductive Health and the Expanded Programme for Immunization (EPI) is being used to increase geographical access and to achieve at least 4 ANC visits for each pregnant woman. Although the uptake of IPT2 has improved from 13% to 58.2%, there is variation in utilization levels in ANC visits; ANC 1 at 73.4%, ANC 2+ at 63.9% and ANC 4+ 21.0% [20].

#### Malaria case management

Effectual case management, consisting of definitive diagnosis and prompt treatment with appropriate anti-malarials, is a key strategic intervention for malaria control. In South Sudan, the malaria diagnosis policy recognizes microscopy as the gold standard for parasitological confirmation. However, due to constraints in human resource and institutional capacity microscopy is mostly restricted to hospital and primary health care centre (PHCC) levels. Malaria rapid diagnostic tests (RDTs), First Response Malaria HRP-2®, remain frontline confirmatory tools at the primary health care unit (PHCU) level and to lesser extent at the hospital and PHCC levels. With only 40% of the health facilities capable of offering definitive diagnostic services [21], most malaria cases are diagnosed clinically with confirmatory diagnosis accounting for 27% only [8].

Resistance to CQ and SP in the country was detected between 2001 and 2004 [13], limiting the efficacy of these monotherapies. In 2005, the policy for treatment of uncomplicated malaria was changed to artemisinin based combination therapy (ACT). Artesunate plus amodiaquine in a co-packaged blister pack, as the first-line malaria treatment. Artemether plus lumefantrine being second-line treatment and quinine third-line [22]. In the 1st trimester of pregnancy and in children below two months, quinine is the recommended treatment. A country-wide roll-out of training of health workers in all the health facilities was conducted between 2007 and 2010. Following the endorsement by the WHO and the successful pilot by IRC in Ganyiel in 2008 with 80% reduction in child mortality [13], Home Management of Malaria (HMM) was implemented under the integrated community case management (ICCM) fashioned around community IMCI principles. In 2009 HMM programme was rolled out to further increase access to ACT.

The recommended treatment for severe malaria is parenteral artesunate with parenteral artemether and parenteral quinine as alternatives. The parenteral anti-malarials are given for at least 24 hrs after which an oral medication (ACT or quinine) would be given to complete treatment if the patient is able to take medication orally. The pre-referral treatment for severe malaria is rectal artesunate along with supportive treatment; tepid sponging, sucrose, and analgesics. Public-Private Partnership mechanisms have been put in place to enable the private sector to conform to national malaria treatment policies and guidelines [22].

#### Monitoring and evaluation of malaria

The NMCP collects routine morbidity case data from all health facilities monthly based on the national HMIS and the IDSR. There exist marked variations in reported malaria cases probably due to gross under - or over -estimations resulting from weak reporting systems (Figures 2 and 3). Confirmed malaria case data are used to assess the progress in diagnosis and treatment and the effect of interventions on malaria. The numbers of malaria cases and deaths reported have varied with times (Figures 4 and 5). With only two members of staff assigned to monitoring and evaluation (M and E), this component remains a major challenge for the NMCP.

Longitudinal measurement of progress in malaria control interventions has been provided by the South Sudan House Hold Survey (SHHS) [23] and the Malaria Indicator Survey (MIS) [8]. The SHHS provides comprehensive surveys every five years based on representative household samples, providing estimates of a range of health and demographic indicators. The SHHS have been conducted in 2006 and 2010 with malaria indicators including; ITN ownership (2006 and 2010) and ITN use (2006 only), coverage of IPTp (2010 only), and nature of treatment of childhood fevers [23,24]. In November 2009 the first MIS collected data for core malaria indicators; coverage of ITNs, IRS, IPTp, and ACT including markers for anaemia and parasite species prevalence [8]. Several population-based surveys, with variation in weight, have been conducted during the life of the 2006–2013 NMCP strategic plan. While the indicators fall far short of the 60% target for 2013, overall there have been some improvement in performance (Table 2).

**Table 2:** Progress in implementation of NMCP strategic plan 2006 - 2013

**Major specific targets for malaria control to be achieved by 2011**						
**Indicator**	**South Sudan household and health survey (2006) [**[21]**]**	**Malaria indicator survey (2009) [**[8]**]**	**South Sudan household and health survey (2010) [**[22]**]**	**Lot quality assurance sampling (LQAS) community based survey (2011) [**[18]**]**	**2011/2012 EPI coverage survey [**[19]**]**	**Target**
**Vector control**						
Proportion of households with at least 1 ITN	11.6%	53.0%	34.2%	40.7%		**80%**
Proportion of children under 5 years who sleep under ITN	27.6%	25.0%		31.2%	40.7%	**60%**
Proportion of structures protected through IRS		2.1%				**80%**
**Case management**						
Proportion of children under 5 years of age with fever who received antimalarial treatment according to the national treatment guidelines within 24 hours of fever onset	2.6%	11.0%		15.6%	39.6%	**60%**
**Malaria in pregnancy**						
Proportion of pregnant women sleeping under ITN		39.0%		29.4%	38.2%	**60%**
Proportion of pregnant women attending ANC who received at least 2 doses of IPT during their last pregnancy		13.0%	51.2%	23.7%	58.7%	**60%**

*NB* The four different surveys had different weights hence the difference in outcomes for those conducted in the same year.

Under the financial and technical auspices of the WHO, 32 malaria sentinel sites with at least 3 per state have been set up and operationalized covering all 10 states across the country. At each sentinel health facility, tracked indicators include: Number of uncomplicated malaria cases; number of severe malaria cases; malaria case fatality rates; number of uncomplicated malaria cases treated with first line anti-malarial; percentage of pregnant women who receive two doses of SP for IPTp; Blood smear and RDT positivity rates; severe anaemia rates and blood transfusion rates among children under five years of age; including stock levels and consumption rates for antimalarial – ACT, SP, injectable quinine, injectable artesunate. With recent funding from USAID, sentinel site surveillance is being strengthened. Malaria drug efficacy monitoring has been conducted from 2001 to 2003 by the MSF [13]. Follow up surveys are underway for 2013 as a collaborative effort between the NMCP, WHO and MSF [16]. Malaria vector bionomics, transmission intensity and insecticide resistance monitoring are also being conducted.

#### Coordination and support for malaria control

The NMCP in South Sudan started in 2004 as part of the Secretariat of Health based in Nairobi, Kenya. Since the signing of the CPA in 2005, major milestones in the fight against malaria have been achieved (Table 2). The NMCP is mandated to control and prevent malaria morbidity and mortality and to minimize the inherent negative social and economic impact country-wide. The objectives are to deploy a scaled-up integrated package of effective malaria control interventions and to promote positive behaviour change for enhanced uptake of interventions. The NMCP spearheads malaria control through policy formulation, quality assurance, coordination of health research, and M and E of performance.

Only one Malaria Strategic Plan (2006–2011) with extension to 2013 has been implemented in South Sudan [10]. Under the leadership of the Programme Manager, the NMCP is organized along five main units; case management unit, vector control unit, Behaviour Change Communication (BCC) unit, M and E unit, and finance and administration unit. At the State MoH, Malaria Coordinators and M and E Officers, are responsible for malaria control. Implementation at the lowest level is through the formal health care delivery system stratified into hospitals and health centres [4]. Overall coordination with other organizations is achieved through the national Malaria Technical Working Group and specific thematic groups for each strategic intervention.

The major funders of the malaria control in South Sudan are the Global Fund, WHO, UNICEF and USAID. Other contributors include the World Bank, MSH, DFID, PSI, Malaria Consortium and CIDA [13]. The partners have contributed both full time staff and provided technical assistance to support the full functionality of malaria control. While the NMCP is striving to uphold “the three-ones” concept: one coordinating mechanism, one implementation plan and one monitoring plan, coordination of malaria partners remains a daunting task.

### Challenges to malaria control in south Sudan

The decades of war in South Sudan virtually led to the collapse of the entire health system, as evidenced by the poor health outcome indicators of the country that are among the worst globally [4]. The situation is aggravated by an increase in population due to refugees, returnees and internally displaced persons. Accordingly, the country experiences exceedingly high malaria transmission intensities with inherent high morbidity and mortality rates [8]. However, epidemiological data concerning malaria morbidity and mortality remains inadequate particularly in rural settings.

Amidst the austerity budget announced by government, humanitarian aid providers i.e. UN agencies, IOM and NGOs continue to play a pivotal role in malaria control. In 2013 about 131,990 conflict-related displacements occurred in South Sudan. These often over-clouded and mobile populations create a major stumbling block to malaria control. The country is not uniformly amenable for malaria control. In the northern states of Northern Bahr el Ghazal (NBeG), Unity and Upper Nile, control is challenged by an influx of South Sudanese returnees (160,000 in 2012 and about 70,000 expected in 2013) and more than 224,000 refugees (39,000 expected by the end of 2013 from Sudan). Lack of access due to either conflict in Unity and Jonglei states or natural disaster like floods in Jonglei, Lakes, NBeG, Unity, Upper Nile and Warrap are also major impediments to control [9].

While technical capacity for exploiting the full potential of IVM exists, financial and human resources to facilitate deployment of a full package of vector control tools are inadequate. There is minimal coordination among partners and a lack of adequate entomological and epidemiological data for rational evidence-based decision making for vector control at state and county levels (Table 3). The functions of the malaria task forces at these levels should be reviewed to strengthen their performance and enhance coordination. Although there is a high political commitment, local funding is non-existent and sustainability is threatened by donor dependency. Mass and routine LLIN distribution campaigns are inconsistent and BCC materials remain minimal. Household ownership, and more importantly, the use of LLINs by vulnerable groups could not reach the required 80% coverage to provide vector control benefits [8,20,21]. Though, 9.0 million LLINs have been distributed to date only 53% of them will still be effective three years post distribution by the end of 2013 [13].

**Table 3:** SWOT analysis of the malaria control programming in South Sudan

**Strengths**	**Weaknesses**
• Strong government leadership, political commitment and advocacy for malaria control.	• Minimal government/domestic funding for malaria control and over dependency on donor funding.
• Presence of active multi-sectoral (UN agencies, NGOs/FBOs) national MTWG and thematic groups led by the NMCP.	
	• Storage of malaria commodities at the central and facility levels are in adequate.
• Availability of policies, guidelines and strategic plans for malaria control and prevention.	• Weak partner linkage and coordination for malaria control at state and county levels.
• A national drug regulatory authority has been inaugurated.	• Inadequate skilled personnel for all aspects of malaria control and frequent staff turnover at all levels.
• Pharmaceutical management TWG to quantify and procurement of WHO prequalified malaria commodities.	
	• There are no appraisal systems to document non performance and also to motivate those that are performing well.
• Adoption and roll pout of HMM as part of the ICCM.	• Lack of quality assurance and control for malaria commodities and equipment.
• Funding from GFATM and other partners to scale malaria interventions.	• Weak communication system and infrastructure with irregular supervision and feedback mechanisms.
• Availability of capacity to conduct operational research for vector and drug resistance.	
	• Lack of public health reference laboratory infrastructure and services at central level.
• Good mass media in the country to facilitate health education, promotion and BCC/IEC.	
	• Limited package and low coverage and utilization of proven malaria vector control tools to attain universal coverage.
• Availability of information sources: HMIS, IDSR, MIS, LQAS and SSHHS.	
• Functional sentinel sites for monitoring and surveillance to regularly guide decision making.	• Minimum entomological data to guide evidence-based deployment of tools.
• Adoption of IVM strategy as a platform for vector control in the country.	• Limited technical support, guidance and coordination on health promotion, BCC and IEC.
	• Constrained health system that may not cope with added pressures of a national programme expansion.
	• Limited definitive diagnosis, frequent stock outs of commodities and unregulated private sector.
**Opportunities**	**Threats**
• Availability of high donor funding to support scale-up of interventions.	• Reducing government financial commitment.
	• Resistance of malaria parasites and vectors to anti-malarials and insecticides respectively.
• Active RBM partnership and large net work of NGOs and private sector to support malaria programming.	
	• Sustainability of funding.
	• Insecurity and inaccessibility.
• Recently established food and drug authority to regulate and facilitate quality control.	• Increasing populations and availability of displaced populations.
• High technical assistance support.	• Influx of untreated nets and abuse/misuse of nets.
• Great potential for higher-level political support.	• Lack of adherence to national treatment guidelines by the private sector clinics and pharmacies.
• Increasing partner commitment and collaboration to establish an entomological laboratory and operations research.	
	• Low levels of literacy.
• The IVM strategy allows for deployment of additional tools and integration with other vector-borne diseases.	• Uncoordinated supply of commodities, availability of fake drugs and unregulated donations of drugs.
• Availability of electronic and print media and coverage of mobile phones and community FM radio stations to support BCC/IEC.	• Weak overall health systems.
	• Limited research and academic institutions with requisite infrastructure to support malaria research.
• Communities that are willing to be key partners in operations and planning for successful outcomes.	

Despite the introduction of ACT as fixed dose at health facility and community level, access to diagnosis and treatment remains a constraint due long distances to health facilities, lack of functional microscopes, and stock-outs of RDTs and anti-malarials. Although progress has been made in ICCM, coverage and coordination remains minimal. The situation is further aggravated by challenges of ensuring correct practices in the largely unregulated private sector coupled with unavailability of treatment guidelines and algorithms. Most malaria cases are treated based on clinical suspicion due to limited diagnostic capacity. The parasites have developed resistance to CQ and SP. Currently there exists an uncoordinated supply chain operated by multiple partners. Procurement and supply chain management is further compromised by the non-existence of accurate consumption data on malaria commodities and the weak pull-based distribution system. The state of most storage facilities does not meet prescribed basic standards.

There is insufficient qualified staff for M and E at all levels of the health system. Most health facilities have poor infrastructure and frequently experience stocks out of key supplies. They lack adequate reporting tools and health worker skills thus compromising quality and resulting into poor data recording and delayed or non -reporting. The county and state levels have limited capacity for supportive supervision and analysis and interpretation of data collected from the health facilities to provide timely corrective feedback. The national level has inadequate funding, lack a database and transport to allow implementation of the plans and to support the lower level structures. The MIP component is the least developed aspect of malaria control in South Sudan and is characterized by lack of access to and late attendance of pregnant women at ANC services. Unavailability of national training modules including limited research on prevention and treatment of MIP present an additional challenge.

Coordination with other departments within the MoH and partners is weak leading to duplication of activities, inequitable distribution of partner support and difficulties in collection and collation of information. This has limited effective harmonization and sharing of messages and materials for BCC, minimal involvement and commitment of community leaders, private sector and government line ministries. As such, the target audience’s uptake of recommended practices to prevent and treat malaria remains low. There is limited human resource capacity with no clear structure at state and county level for malaria control. The minimal financial and technical capacity and high donor dependency has resulted in limited Government funding and inadequate support for operations (Table 3).

## Discussion and evaluation

Following the call by the WHO for scaled-up malaria control efforts [25], coupled with unparalleled availability of resources, targets for control and elimination have been established [26-28]. In response to the huge burden of malaria in sub-Saharan Africa, endemic countries are implementing an integrated approach to malaria control. However, effective malaria control in post-conflict settings is hampered by a multiplicity of challenges. In South Sudan the signing of the CPA and the attainment of independence has facilitated for improvement in health service delivery and operational malaria control [10].

The country embraces the WHO recommendations on effective malaria control including; use of ACT for treatment of uncomplicated malaria, definitive diagnosis by light microscopy and RDTs [22], and vector control within the context of the IVM strategy [18]. Despite the progress in scaling-up interventions, malaria resurgence was confirmed by an incremental annual trend in malaria cases with a concomitant increase in deaths. Routine surveillance data of *P. falciparum* malaria from 2003 through 2008 showed a precipitous decline and a steady re-emergence through 2012 (Figures 4 and 5). Health facility reporting may have fluctuated during these times of turmoil, even after the CPA, and this would greatly confound any real change in malaria prevalence over time. Consequently, health facility based reporting is likely to be of such low quality as to be of limited value. By December 2012, only 68.8% of facilities routinely reported on malaria [13]. The available data likely underestimate the actual number of cases because healthcare providers do not always provide complete reports and many patients never visit health facilities. From 2009 to 2012 there was a gradual decrease in the number of ITNs and a concomitant gradual increase in the number of cases and deaths due to malaria in health facilities. There is need for increased mass distribution of ITNs and maintaining high coverage through supplementary distribution mechanisms i.e. continuous distribution and routine facility based distribution.

Before the signing of the CPA, data collection and reporting was under direct support and supervision of UNICEF/WHO in South Sudan. After the CPA, the responsibility to run the health services reverted to the MoH that was grappling with infrastructure and human resource challenges, resulting in a drastic decline (2006–2008) in reporting. Parasitological confirmation before treatment with antimalarials increased from 27% in 2009 to 40% in 2011 [13]. Therefore, strengthened health service delivery and improved HMIS/DHIS reporting system could in part explain the observed upsurge in annual reported cases from 2008 to 2012. The weekly IDSR currently reports on suspected malaria cases and deaths with over 55% of functioning health facilities and a timeliness of 70% (8). Weekly surveillance bulletins, that highlight malaria reporting performance and outbreak alerts, are produced from reports generated through the IDSR system and circulated to partners [29].

The prevalence has been shown to be higher in the Greater Equatoria, followed by the Greater Bahr el Ghazal and lower in the Greater Upper Nile Regions of South Sudan [8]. There is great heterogeneity in the number of reported annual malaria cases by month and by state (Figures 2 and 3). In the past four years the highest malaria cases were reported in 2012 with CES leading followed by WES. In 2011, NBeG, WBeG, WES and EES recorded the highest cases, while UNS, WES, WRP, CES and Jonglei were the most affected in 2012. The higher cases in CES and WES is due to their location in higher transmission zone. In UNS, WRP and NBeG the high transmission could be ascribed to the recent heavy flooding [9]. Although LLIN distribution has been the main vector control intervention, the intervention falls short of its efficacy due to misuse, low coverage and utilization due to community practices such as sleeping outdoors, fishing and fencing. Routine distribution for pregnant women and children less than five years is low. BCC/IEC remains minimal thus affecting uptake. Due to harsh conditions their insecticide and physical durability is compromised. As housing infrastructure is becoming more amenable for IRS in urban areas, the intervention should be prioritized in these settings. About 4.7 million LLINs have been distributed through mass campaigns in 2013. Continuous distribution has been piloted in South Sudan and plans to scale up the mechanism are underway.

Effective deployment of conventional key malaria vector control interventions is mostly challenged in conflict and post-conflict situations. In South Sudan prevention of malaria in pregnant women and children through distribution of ITNs, IPT, RDTs and treatment with ACT has been prioritized including ICCM. With the increasing influx of mobile displaced populations, humanitarian groups have used these strategies for emergency response among refugees and returnees with striking efficacy. However, logistical assessments are critical for the correct quantifications of malaria commodities. Recently Mentor Initiative an NGO has embarked on IRS deployment with remarkable impact on the burden of malaria. This substantiates the premise that IRS is amenable and effective in emergency situations.

South Sudan has one of the highest malaria burdens in sub-Saharan Africa; the disease remains a leading cause of morbidity and mortality in the country [8]. This could be a function of increased intrinsic malaria potential attributable to several factors including; malaria epidemics and more localized outbreaks, environmental factors (e.g. extensive flooding) and climatic changes. Other potential contributors could be; movement of populations with little immunity into areas of high transmission, deteriorated socioeconomic situation, as well as lack of access to effective anti-malaria treatment in some areas [30]. The use of fake anti-malarial drugs could potentially aggravate the malaria re-emergence situation [31]. A clear understanding of the effectiveness of control tools, increased health information and integration of community and health facility malaria reporting are necessary.

Malaria control problems are further compounded by limited supply of health services due to a serious lack of qualified staff; inadequate equipment and supplies; long distances to facilities, poor roads and transport; dysfunctional referral system and cultural and financial barriers. Presently, only 44% of the population has access to health services within 5 km walking distance in South Sudan [13]. The presence of a multiplicity of humanitarian groups in this post-emergency setting presents a unique opportunity to integrate efforts and optimize the utilization of the limited available human and financial resources. To mitigate duplication of efforts by multiple partners operating in the same geographical areas, implementing partners have been allocated specific states for support; 1) World Bank through IMA: Eastern Equatoria, Unity State, Lakes state, Warrap State, Western Bahr el Ghazal and Northern Bahr el Ghazal; 2) USAID through JHPIEGO: Central Equatoria and Western Equatoria and 3) DFID through Crown Agents: Jonglei and Upper Nile. In conflict or post-conflict situations basic health service delivery is assumed by international donors, NGOs and FBOs. As the situation stabilizes, efforts should shift from fragile top-bottom systems centered on emergency relief by humanitarian groups to more sustainable development systems managed by the government.

While appreciable progress has been made relative to the 2006–2013 malaria control strategic plan (Table 2), the country has experienced challenges unique to the region [32]. There is persistently high levels of transmission coupled with inadequate health care resources that is likely to decrease due to donor fatigue; weaknesses in the health system with a fragmented malaria community and poor coordination; a lack of detailed understanding of malaria epidemiology and impact of interventions and optimal use of control tools; inappropriate case management and inadequate utilization of drugs in malaria prevention; inadequate epidemic preparedness and response, and; potential of increasing drug and insecticide resistance [32].

Cognizant that resistance to anti-malarial drugs is a major public health problem, which potentially hinders effective malaria control, a surveillance system has been set up to facilitate monitoring and containment of this phenomenon [16]. Drug efficacy studies demonstrated resistance to SP, which remains a drug of choice for MIP [13]. Studies are on going to determine the resistance levels of ACT in selected areas of the country. Preliminary data on insecticide resistance demonstrated tolerance to DDT and pyrethroids [17]. However, the study did not characterize the *Anopheles* mosquitoes to species level. There is need for extensive studies to establish the insecticide resistance profiles of malaria vectors in the country.

Malaria monitoring, evaluation and surveillance are essential for establishing the effectiveness of interventions and early detection of, and prompt response to malaria outbreaks and epidemics. In South Sudan, the reporting system for malaria diagnosis and treatment is fully integrated into the routine health information systems. Improved quality routine health facility data has proved useful in assessing the impact of malaria control measures on the incidence of severe malaria in Africa [33], malaria cases and deaths in all age groups [34-36] and has facilitated for improved spatial mapping of malaria trends for local programme monitoring and resource planning [37]. As such the use of routine surveillance data in determining the temporal effects of malaria control is important for monitoring and evaluation [38]. This requires overcoming challenges over timeliness of data collection, management and reporting and use at county and health facility level to inform decision-making. Therefore, improved infrastructure and strengthened human resources are critical for quality malaria routine surveillance in South Sudan. Capacity building has been embarked upon with trainings conducted in malaria epidemic surveillance and therapeutic efficacy testing, malariology, malaria management and planning, insecticide resistance monitoring, malaria microscopy and quality assurance, malaria case management and prevention, including malaria sentinel surveillance.

More than 80% of the national malaria strategic plan has been funded through external sources. Up to 170 million USD has been secured through consolidated Global Fund rounds 7 and 10 malaria grants for the period of 2008–2016 with 118.5 million USD already disbursed for provision of anti-malarials, RDTs, LLINs and programme management support. Other partners; USAID, DFID, UN agencies (WHO and UNICEF) and various NGOs have also contributed considerably. Apart from 13 million USD allocated to malaria control in 2007/8 under the Multi Donor Trust Fund, domestic funding for health including malaria control has not been significant and has steadily dropped from 7.9% in 2006 to 4.2% in 2010 [13].

The health sector in South Sudan requires substantial technical, programmatic, managerial and financial input and investment. To move out from humanitarian assistance into development country-level sustainable programming and ensure allocation of adequate local funding, malaria control in South Sudan is prioritized under the Basic Package of Health Services (BPHS) which provides the operational reference for the implementation of the Health Policy and HSDP for the period 2011–2015. The BPHS is a key document for all stakeholders and is the platform for the cooperation between the implementing and capacity building partners. The HSDP is closely linked to the health section in the social and human development pillar of the South Sudan Development Plan (SSDP). This policy framework for health service delivery would increase advocacy and convince donors and parliament members to fund malaria control when faced with food insecurity and the necessity of meeting other essential needs, such as shelter and economic livelihoods for the thousands of returnees and those currently displaced.

Prospects for effective malaria control and elimination in South Sudan are huge (Table 3). However, more comprehensive and sustained control measures will likely be required to begin to decrease the massive malaria burden. These would include; Strengthened BCC, confirmation of outbreaks, epidemic preparedness and response and PSM for malaria commodities; communication systems and infrastructure; regular supervision and feedback mechanisms; human and technical capacity building; improvement in quality assurances and control. A full packaged IVM approach, including evidence-based decision-making; integrated approaches; collaboration within the health sector and with other sectors; advocacy, social mobilization, and legislation; and capacity-building is required [39]. To do all this, allocation of adequate local financial resources would be critical.

## Conclusions

South Sudan has dealt with constant threats from an influx of returnees, refugees, IDPs, flooding, and civil strife. Given the trend and magnitude of the malaria burden in the country a more defined malaria control strategic direction will be critical. To improve services in a post-conflict setting with, elements of conflict and severe resource limitations, there is need to address the increasing malaria case load through deployment of interventions that are amenable to the local situation and improve case detection, data analysis and reporting. A clear understanding of the effectiveness of control tools and improved health information system with integration of community is necessary. All this calls for improved requisite infrastructure and strengthened human and financial resource capacity in South Sudan.

## Abbreviations

ACT: Artemisinin-based combination therapy; AS: Artesunate; AQ: Amodiaquine; CES: Central equatoria state; CPA: Comprehensive peace agreement; DHIS: Demographic health information survey; EES: Eastern equatoria state; EPI: Expanded programme for immunization; FBO: Faith-based organizations; GoSS: Government of southern Sudan; HMM: Home management of malaria; HMIS: Health management information system; HRP-2: Histidine rich protein-2; HSDP: Health sector development plan; HSSP: Health sector strategic plan; ICCM: Integrated community case management; IDSR: Integrated disease surveillance response; IMCI: Integrated management of childhood infections; IMA: Interchurch medical assistance; IOM: International organization for migration; IPT: Intermittent preventive treatment; IRS: Indoor residual spraying; LLINs: Long-lasting insecticidal nets; MDGs: Millennium development goals; MIP: Malaria in pregnancy; MIS: Malaria indicator survey; MoH: Ministry of health; MPR: Malaria programme review; MSF: Médecins Sans Frontières; NBeG: Northern Bahr el Ghazal; NGOs: Non-governmental organizations; NMCP: National malaria control programme; OPD: Out-patient department; PHCC: Primary health care centre; PSM: Procurement and supply chain management; RBM: Roll back malaria; RDTs: Rapid diagnostic tests; RSS: Republic of south Sudan; SHHS: Sudan house hold survey; SP: Sulpadoxine-pyrimethamine; UNICEF: United nations international children emergence fund; UNS: Unity state; USAID: United States agency for international development; WBeG: Western Bahr el Ghazal; WES: Western Equatoria state; WHO: World health organization; WRP: Warrap state.

## Competing interests

The authors declare that they have no competing of interests.

## Authors’ contributions

HP: Managed the NMCP in South Sudan. MJ, AJ and CD: Coordinated deployment of malaria control interventions. BS, MY: monitored and evaluated the interventions. SPB: Collaborated. EC: Conceived the idea and wrote the paper. All authors read and approved the final manuscript.
